# Delineation of Natural Killer Cell Differentiation from Myeloid Progenitors in Human

**DOI:** 10.1038/srep15118

**Published:** 2015-10-12

**Authors:** Qingfeng Chen, Weijian Ye, Wei Jian Tan, Kylie Su Mei Yong, Min Liu, Shu Qi Tan, Eva Loh, Kenneth TE Chang, Thiam Chye Tan, Peter R. Preiser, Jianzhu Chen

**Affiliations:** 1 Humanized Mouse Unit, Institute of Molecular and Cell Biology, Agency for Science, Technology and Research (A*STAR), 138673, Singapore; 2 Interdisciplinary Research Group in Infectious Diseases, Singapore-Massachusetts Institute of Technology Alliance for Research and Technology, 138602, Singapore; 3 School of Biological Sciences, Nanyang Technological University of Singapore, 637551, Singapore; 4 Department of Obstetrics & Gynaecology, KK Women’s and Children’s Hospital, 229899, Singapore; 5 Department of Pathology and Laboratory Medicine, KK Women’s and Children’s Hospital, 229899, Singapore; 6 Duke-NUS Graduate Medical School, 169857, Singapore; 7 The Koch Institute for Integrative Cancer Research and Department of Biology, Massachusetts Institute of Technology, Cambridge, MA 02139, USA

## Abstract

Understanding of natural killer (NK) cell development in human is incomplete partly because of limited access to appropriate human tissues. We have developed a cytokine-enhanced humanized mouse model with greatly improved reconstitution and function of human NK cells. Here we report the presence of a cell population in the bone marrow of the cytokine-treated humanized mice that express both NK cell marker CD56 and myeloid markers such as CD36 and CD33. The CD56^+^CD33^+^CD36^+^ cells are also found in human cord blood, fetal and adult bone marrow. Although the CD56^+^CD33^+^CD36^+^ cells do not express the common NK cell functional receptors and exhibit little cytotoxic and cytokine-producing activities, they readily differentiate into mature NK cells by acquiring expression of NK cell receptors and losing expression of the myeloid markers. Further studies show that CD33^+^CD36^+^ myeloid NK precursors are derived from granulo-myelomonocytic progenitors. These results delineate the pathway of human NK cell differentiation from myeloid progenitors in the bone marrow and suggest the utility of humanized mice for studying human hematopoiesis.

Natural killer (NK) cells are a key innate immune cell type with diverse functions. NK cells were originally discovered for their ability to kill tumor cells and non-self cells without prior stimulation^1^. Since then, they have been shown to play an essential role in immediate responses to infections and in activation of the adaptive immune responses. NK cells exert their diverse functional effects through direct cell-cell contact and secretion of cytokines such as interferon γ (IFN-γ) and tumor necrosis factor α (TNF-α)^2^. In humans, NK cells are usually identified by their expression of CD56 in the absence of CD3^3^.

Studies have shown that NK cells can be differentiated from both lymphoid and myeloid progenitors. In mice, adoptive transfer of Lin^-^IL-7R^+^Thy-1.1^−^Sca-1^low^c-Kit^low^ common lymphoid progenitors (CLP) into irradiated recipients gives rise to the donor-derived T, B and NK cells in about 4 weeks^4^. Commitment of CLP towards NK cells differentiation is associated with expression of CD122 and the ability to differentiate into mature NK cells, but not T, B and myeloid cells, *in vitro*^5^. In humans, purified CLP, CD34^+^CD38^+^CD10^+^CD45RA^+^, give rise to T and B cells as well as NK cells^6^. In c-Kit^W/W^ mice, CLP is severely depleted but NK cells and myeloid lineage cells are not affected^7^,^8^. These observations support the possibility of NK cell development from myeloid precursors. Consistently, *in vitro*, purified CD34^+^CD38^+^CD123^low^CD45RA^−^ human common myeloid progenitors (CMPs) can give rise to CD56^high^ NK cells in the presence of murine stromal cells and IL-15^9^. Furthermore, human cord blood (CB) contains a population of CD56^+^CD33^+^ NK cells that have lower cytotoxic activity, suggesting their immature status^10^. Based on CD34, CD117 and CD94 expression, human NK cell maturation in the secondary lymphoid tissues can be divided into four stages and CD33 expression is detected in the first three stages, but is lost upon maturation into CD56^high^ NK cells^11^.

Despite these advances, our understanding of NK cell development in humans is still incomplete. To date, most studies on human NK cell development rely on *in vitro* differentiation in cell cultures and further validation in rodent models. However, *in vitro* cultures may not mimic the complex *in vivo* physiological conditions, such as the interaction networks among various cell types and organ-specific feature of NK cells^12^. There are also significant differences between human and mouse NK cells. Most notably, mouse NK cells do not express CD56 and some activation and inhibitory receptors such as NKp30, NKp44, and KIR. Human and mouse NK cells also differ significantly in signal transduction and activation^13^. Thus, the study of human NK cell development requires better *in vivo* models.

Reconstitution of human immune cells in immunodeficient mice following engraftment of human hematopoietic stem/progenitor cells (humanized mice) appears to provide a system to study human NK cell development under physiological conditions. In particular, we have shown that human NK cell reconstitution in the recipient mice can be greatly enhanced following expression of human cytokines IL-15 and Flt-3/Flk-2 ligand (Flt-3L)^14^. Here, we show that while gene expression profiles of human CD56^+^ NK cells from spleen, liver and lung of humanized mice are similar, that of CD56^+^ NK cells from the bone marrow (BM) exhibit significant differences. Further investigations show that the differences are because most of CD56^+^ cells in the BM are immature NK cells. Interestingly, the immature NK cells also express myeloid markers such as CD33 and CD36 that are usually found on monocytes/macrophages, platelets and megakaryocytes, but not mature NK cells^15^. The CD36^+^CD33^+^ immature NK precursors are also found in human CB, fetal and adult BM. We further show that these myeloid NK precursors can be derived from granulo-myelomonocytic progenitors (GMPs), and give rise to mature NK cells. These findings further delineate the pathway of human NK cell differentiation from myeloid progenitors in the BM and suggest the utility of humanized mice for studying the development of human NK and other immune cell types.

## Results

### Most NK cells in the BM of humanized mice express myeloid markers and are immature

**Figure 1:**
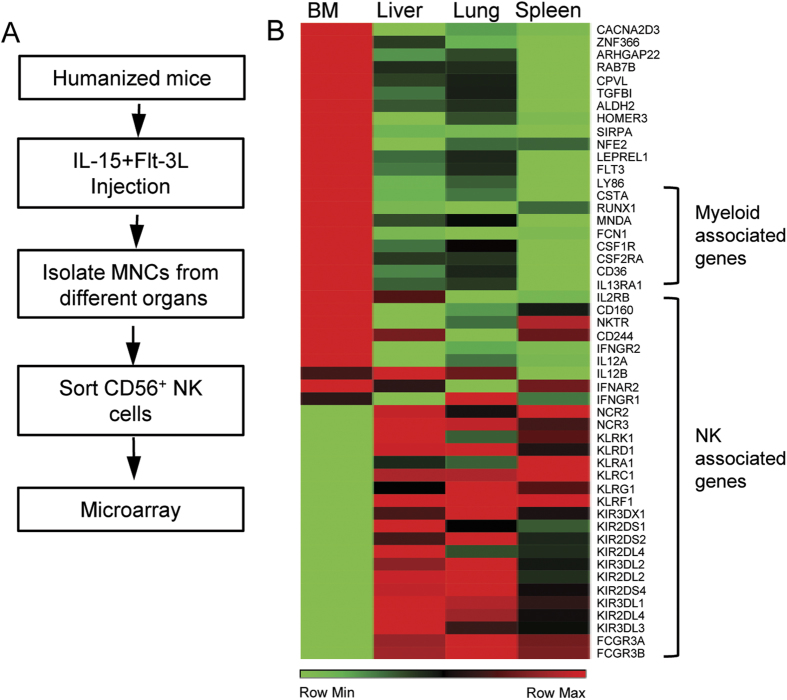
Comparison of transcription profiles of NK cells from different organs of humanized mice. (**A**) Flow of experimental procedure. CD56^+^ NK cells were pooled from five humanized mice reconstituted with the same donor HSCs. (**B**) Hierarchical clustering analysis of transcriptomes among NK cells from BM, spleen, liver and lung.

We have previously shown that expression of human cytokines IL-15 and Flt-3L in humanized mice dramatically enhances human NK cell reconstitution^14^. To further investigate human NK cell development in humanized mice, we carried out transcriptional analysis of CD56^+^ cells from various organs. Specifically, humanized mice with 40% or more human leukocyte reconstitution in the peripheral blood mononuclear cells were injected with plasmids encoding human IL-15 and Flt-3L. Nine days after plasmid injection, mononuclear cells (MNCs) from BM, spleen, liver and lung were prepared and CD56^+^ NK cells were purified by cell sorting. RNA was extracted from the purified CD56^+^ NK cells and analyzed by microarray using Agilent SurePrint G3 Human GE 8 × 60 K Microarray (Fig. 1A). Analysis of microarray data revealed that NK cells from spleen, liver and lung shared similar transcription profiles, whereas NK cells from the BM showed significant differences in gene expression (Fig. 1B). In particular, BM NK cells were enriched for myeloid lineage marker expression, including CD33 and CD36, while the expression levels of NK cell functional receptors such as NKG2D, NKG2A and NKp46, were much lower than NK cells from spleen, liver and lung (Fig. 1B).

**Figure 2:**
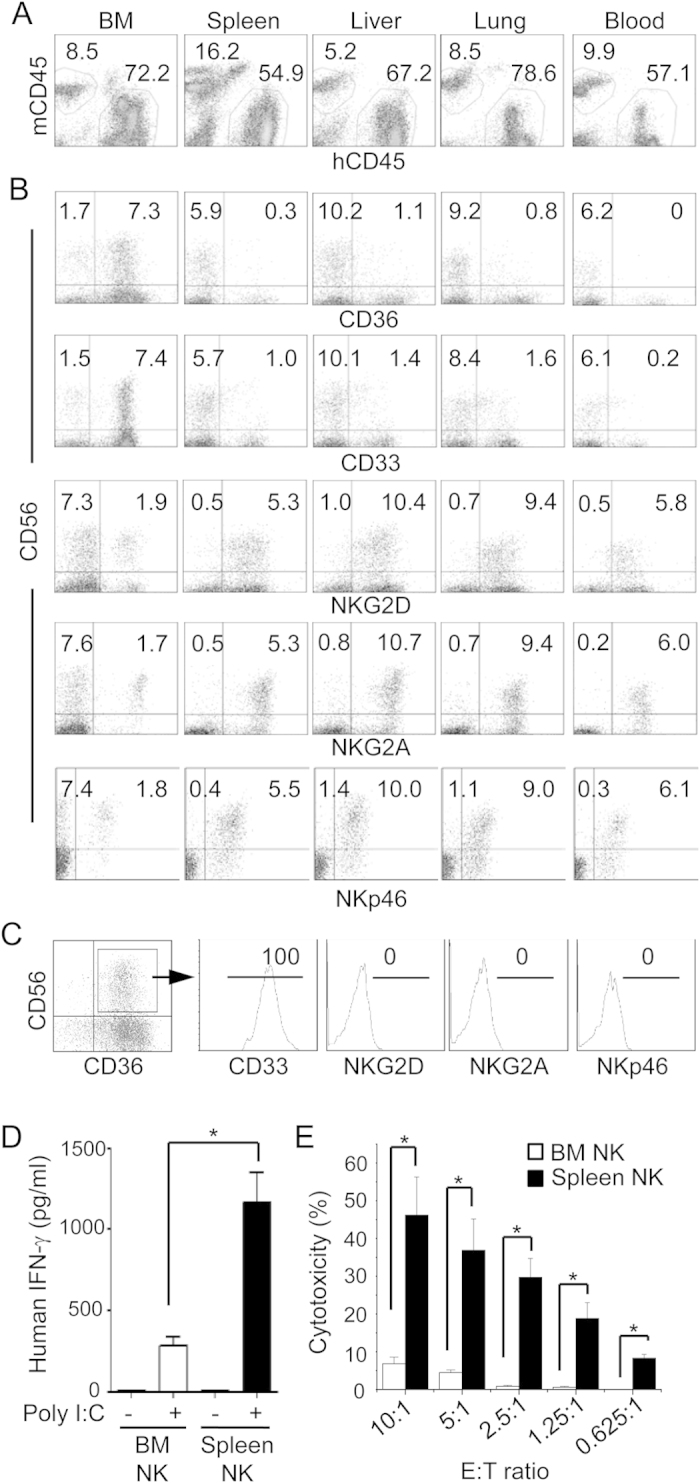
Comparison of NK cell phenotype and function from different organs of humanized mice. Humanized mice were injected with IL-15 and Flt-3L plasmids and MNCs were prepared from the indicated organs 9 days later. (**A**–**C**) MNCs were stained for mouse CD45.1 (mCD45), human CD45 (hCD45), CD56 plus CD33, CD36, NKp46, NKG2A, or NKG2D. Shown are mCD45 versus hCD45 staining profiles of indicated organs gating on live cells (**A**), CD56 versus CD36, CD33, NKG2D, NKG2A and NKp46 staining profiles gating on hCD45^+^ cells (**B**), and histograms of CD33, NKG2D, NKG2A and NKp46 gating on CD56^+^CD36^+^ cells (**C**). Representative data from one of five mice per group are shown. The numbers indicate the average percentages of cells in the gated regions. For lack of space, standard error of mean (SEM) is not shown. The analyses were repeated with three different donor HSCs. (**D,E**) CD56^+^ NK cells were purified from BM and spleen of humanized mice by magnetic cell sorting. Cells (100,000/well) were cultured for 24 hrs in the presence or absence of poly I:C and the level of human IFN-γ was quantified in the culture supernatant (**D**). BM and spleen NK cells were mixed with K562 cells at the indicated effector to target (E:T) ratios for 4 hrs and lysis of target cells was determined by measuring LDH enzymatic activity in the supernatant (**E**). Data shown are mean ± SEM of two separate experiments. *p < 0.05.

To validate the differences in gene expression between NK cells from BM and spleen, liver, lung and blood, we analyzed selected surface markers by flow cytometry. MNCs were prepared from various organs of cytokine-treated humanized mice and stained for mouse CD45 (mCD45), human CD45 (hCD45), and human CD56, CD36, CD33, NKG2D, NKG2A, and NKp46 followed by flow cytometry. A large fraction of MNCs in the BM (64.7 ± 15.2%), spleen (49.5 ± 18.7%), liver (67.7 ± 18.2%), lung (70.9 ± 11.6%) and blood (56.9 ± 16.3%) stained positive for human CD45 (Fig. 2A). Human CD45^+^ cells were then analyzed for CD56 versus CD33, CD36, NKG2D, NKG2A and NKp46 expression (Fig. 2B). In the BM, approximately 80% of human CD56^+^ cells stained positive for CD33 or CD36 whereas less than 20% of human CD56^+^ cells in the spleen, liver, lung and blood were positive for CD33 or CD36. In contrast, only ~20% of human CD56^+^ cells were positive for NKG2A, NKG2D or NKp46 in the BM, whereas in the other organs the percentages were >80% (Fig. 2B). Furthermore, human CD56^+^CD36^+^ NK cells in the BM were positive for CD33 but negative for NKG2D, NKG2A and NKp46 (Fig. 2C). Thus, the majority of human CD56^+^ NK cells in the BM express myeloid markers such as CD36 and CD33 and the CD56^+^CD36^+^ NK cells do not express NK cell markers such as NKG2D, NKG2A and NKp46. Conversely, human CD56^+^ NK cells in the spleen, liver, lung and blood are mostly positive for NKG2A, NKG2D and NKp46 with very few are positive for CD36 and CD33.

When purified CD56^+^ NK cells from BM and spleen were stimulated with polyinosinic: polycytidylic acid (poly I:C), BM NK cells secreted 3 fold less amount of IFN-γ than splenic NK cells (Fig. 2D). Likewise, BM NK cells lysed only ~5% of K562 target cells at effector to target ratio of 10 to 1, whereas cytotoxic activity of splenic NK cells was at least 10 fold higher (Fig. 2E). These data show that most of BM NK cells in humanized mice are functionally immature and phenotypically different from mature NK cells in the peripheral organs. As the CD33^+^CD36^+^ NK cells in the BM also express other myeloid markers (Fig. 1B), we refer them as myeloid NK precursors.

### Myeloid NK precursors are present in human cord blood, fetal and adult BM

**Figure 3:**
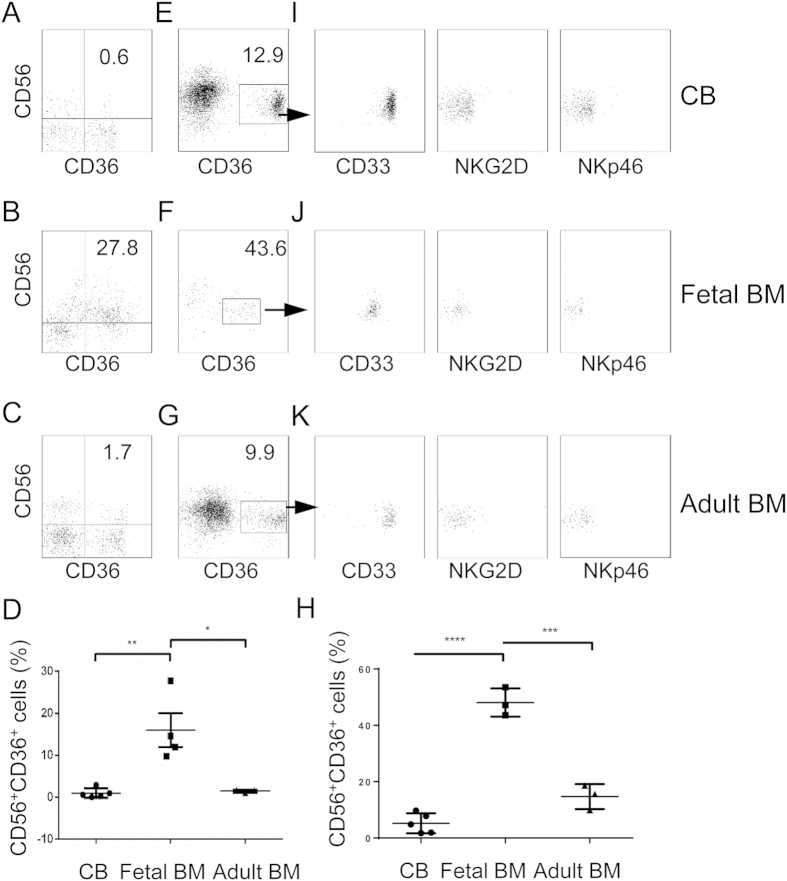
Detection of myeloid NK precursors in human CB, fetal and adult BM. (**A–C**) MNCs were prepared from human CB, fetal and adult BM and stained for CD56 and CD36. Shown are representative CD56 versus CD36 staining profiles of 5 human CB (**A**), 4 fetal BM (**B**) and 3 adult BM (**C**). (**D**) Comparison of percentages of CD56^+^CD36^+^ cells in CB, fetal BM and adult BM. (**E–K**) CD56^+^ cells were enriched from human CB, fetal and adult BM and stained for CD56, CD36, plus CD33, NKG2D or NKp46. Shown are CD56 versus CD36 staining profiles of live cells (**E–G**), Comparison of percentages of CD56^+^CD36^+^ cells after CD56 positive selection of CB, fetal BM and adult BM (**H**) and CD56 versus CD33, NKG2D or NKp46 staining profiles of CD36^+^ cells (I-K) from CB, fetal and adult BM. The numbers indicate percentages of cells in the gated regions. **p < 0.005, *p < 0.05, ****p< 0.0001, ***p < 0.001.

Intrigued by the presence of the myeloid NK precursors in the BM of humanized mice, we sought to determine if such a population also exists in humans. MNCs were isolated from human CB, fetal and adult BM and stained for CD56 and myeloid markers CD33 and CD36. As shown in Fig. 3A–D, a significant population of CD56^+^ NK cells stained positive for CD36 in the fetal BM (16.06% ± 4.04, n = 4), but very few were positive in the CB (0.98% ± 0.51, n = 5) and adult BM (1.49% ± 0.20, n = 3). To better visualize these cells, we first enriched CD56^+^ cells from CB, fetal and adult BM by magnetic selection and then stained for CD36, CD33, NKG2D and NKp46. A distinct population of CD56^+^ cells was positive for CD36 in CB, fetal and adult BM (Fig. 3E–H), although the population was more prominent in the fetal BM (48.1% ± 5.0, n = 3) than in CB (5.2% ± 3.5, n = 5) and adult BM (14.7% ± 4.4, n = 3). All CD56^+^CD36^+^ cells co-expressed CD33 but were negative for NKG2D and NKp46 (Fig. 3I–K), consistent with the observation in humanized mice. Different from humanized mice where the level of CD56 was the same between CD36^+^ and CD36^−^ NK cells in the BM, the level of CD56 was lower on CD36^+^ NK cells than on CD36^−^ NK cells in human CB, fetal and adult BM (Fig. 3E–G). These data show that the myeloid NK precursors are normally present in human: most abundant in fetal BM but less frequent in CB and adult BM.

### Myeloid NK precursors can differentiate into mature NK cells *in vitro*

**Figure 4:**
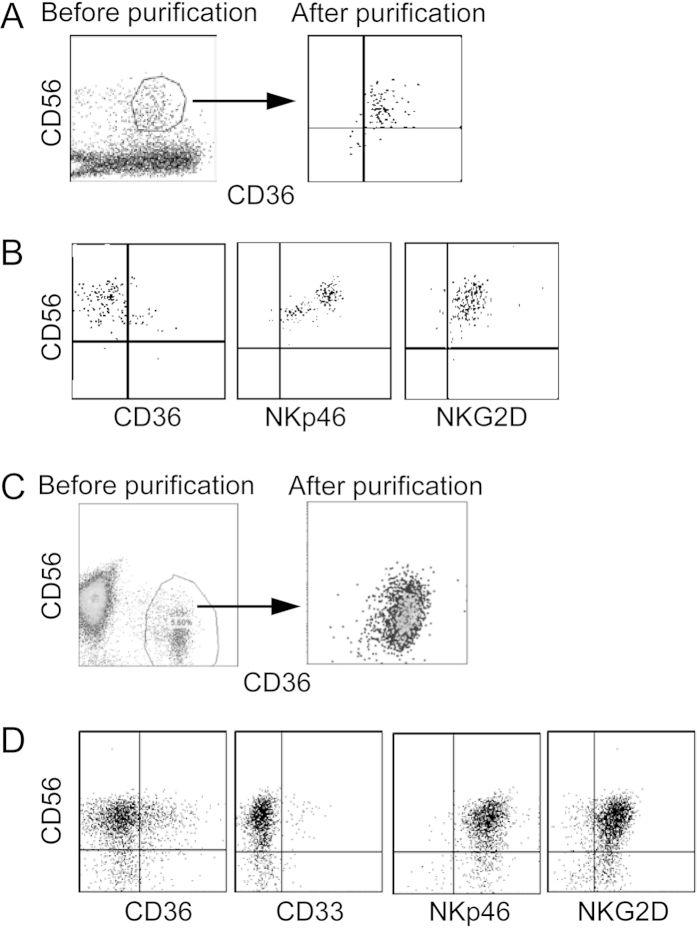
Maturation of myeloid NK precursors *in vitro.* Human CD56^+^CD36^+^ NK precursors were purified from BM of cytokine-treated humanized mice or from CB by cell sorting. Purified cells were cultured in NK differentiation medium and analyzed for CD56, CD36, CD33, NKG2D and NKp46 followed by flow cytometry. (**A,C**) CD56 versus CD36 staining profiles before and after purification from the BM of humanized mice (**A**) or CB (**C**). (**B,D**) CD56 versus CD36, CD33, NKG2D and NKp46 staining profiles 14 days after culture of purified cells from the BM of humanized mice (**B**) and CB (**D**). The experiments were repeated at least 3 times with similar results.

To determine the differentiation potential/capacity of the myeloid NK precursors, CD56^+^CD36^+^ NK cells were purified from the BM of cytokine-treated humanized mice by cell sorting and cultured in differentiation medium containing human stem cell factor (SCF), IL-15 and Flt-3L. Seven and 14 days later, cells were stained for CD56, CD36, NKG2D and NKp46. Associated with the loss of CD36 expression, NKG2D and NKp46 expression was observed (Fig. 4A,B). Similarly, CD56^+^ cells were enriched from human CB and then CD56^+^CD36^+^ cells were purified by cell sorting followed by *in vitro* differentiation as above. After 14 days, most of CD56^+^ cells lost the expression of myeloid markers CD33 and CD36 but became positive for NK cell functional makers NKp46 and NKG2D (Fig. 4C–D). Thus, the myeloid NK precursors are immature and capable of further differentiation into mature NK cells.

### Myeloid NK precursors represent an intermediate stage in human NK cell development

**Figure 5:**
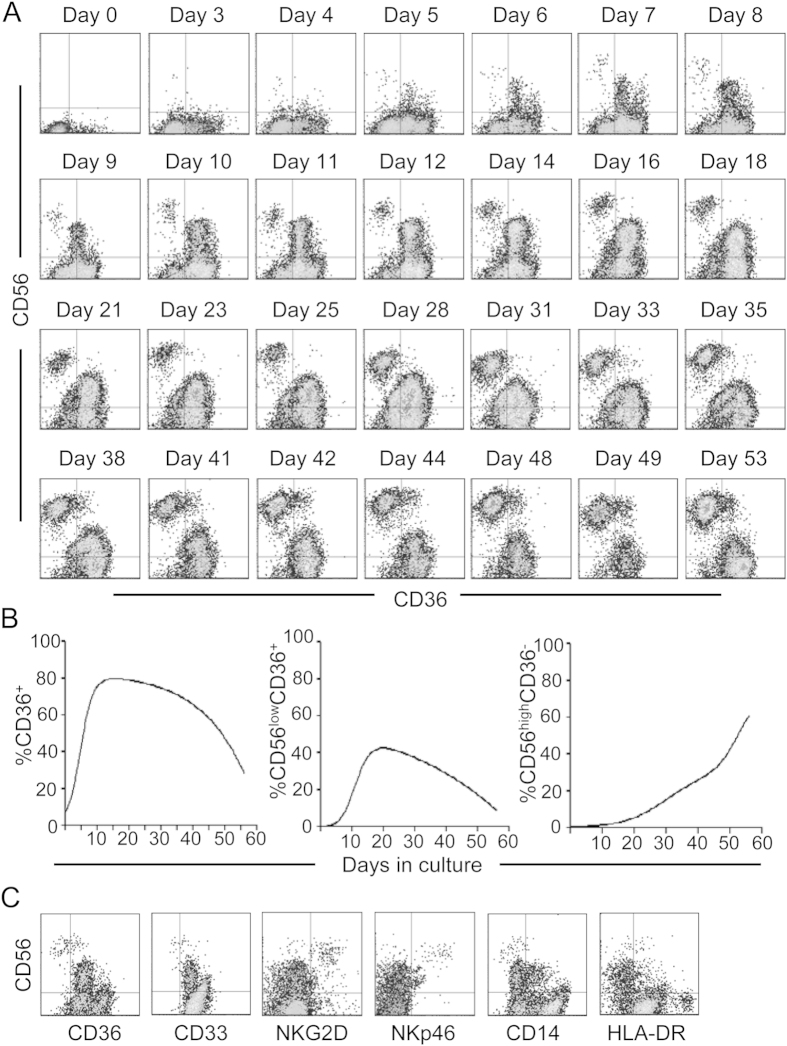
Differentiation of myeloid NK precursors from hematopoietic stem/progenitor cells *in vitro*. CD34^+^ cells were purified from human CB and cultured in NK differentiation medium. Cells in the culture were analyzed by flow cytometry for CD56 and CD36 everyday initially and every two to four days later. Shown are CD56 versus CD36 staining profiles at the indicated time points (**A**) and percentages of CD36^+^, CD56^low^CD36^+^, and CD56^high^CD36^−^ cells over time (**B**). (**C**) Cells in the culture were analyzed for CD56, CD36, CD33, NKG2D, NKp46, CD14 and HLA-DR every 7 days. Shown are staining profiles of CD56 versus CD36, CD33, NKG2D, NKp46, CD14 and HLA-DR at day 14. The experiments were repeated at least 3 times with similar results.

To further determine that the myeloid NK precursors are intermediates during human NK cell development, we employed a widely used *in vitro* culture system to differentiate mature NK cells from CD34^+^ hematopoietic stem/progenitor cells^9^. Human CD34^+^ cells were purified from CB and cultured in NK cell differentiation medium. Cells in the culture were analyzed for CD56 and CD36 expression over time. Prior to culture (day 0), human CD34^+^ cells were negative for CD56 and only a small fraction (<10%) was CD36^+^ (Fig. 5A). Three days after culture, most cells were still negative for CD56 but the fraction of CD36^+^ cells increased. With time, the proportion of CD36^+^ cells increased gradually and then started to decrease 21 days after culture (Fig. 5B). Associated with the increase in CD36^+^ cells, CD56^low^CD36^+^ cells were first detected 5 days after culture, and continued to increase to become a major population around day 10. Starting from 21 days after culture, the proportion of CD56^low^CD36^+^ cells started to decline. CD56^high^CD36^−^ cells became apparent 7 days after culture and the proportion kept increasing and became a significant population by day 14. Furthermore, every 7 days, the cells in the cultures were analyzed for CD33, NKG2D, NKp46, CD14 and HLA-DR. As shown in Fig. 5C, CD56^low^CD36^+^ cells were positive for CD33 but negative for NKG2D and NKp46, whereas CD56^high^CD36^−^ cells were negative for CD33 but positive for NKG2D and NKp46. Most of CD56^low^CD36^+^ and CD56^high^CD36^−^ cells were negative for CD14 and HLA-DR, suggesting that they are not mature myeloid cells. These results show that CD36 and CD33 are transiently expressed during NK cell development, further supporting the designation of these cells as myeloid NK precursors.

**Figure 6:**
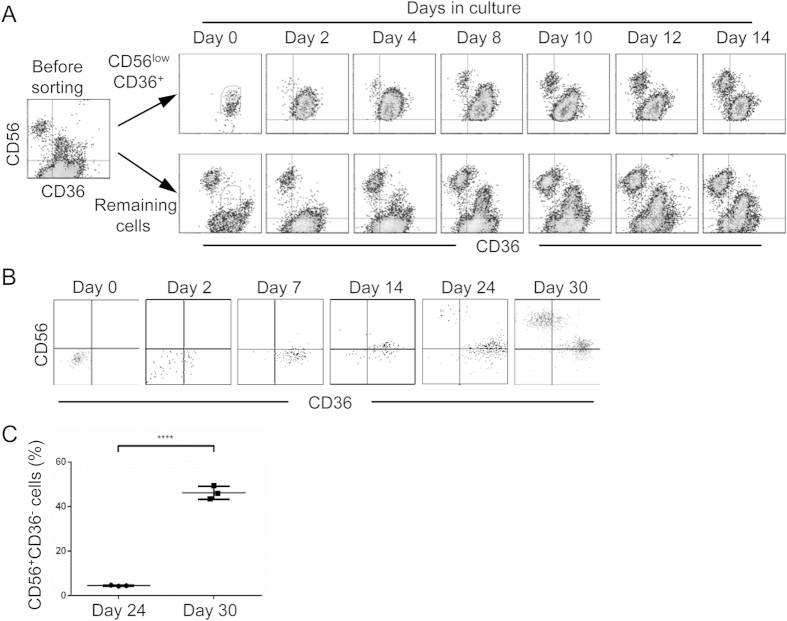
Differentiation of CD56^high^ mature NK cells from CD56^low^CD36^+^ myeloid precursors. CD34^+^ cells were purified from human CB and cultured in NK differentiation medium. Fourteen days after culture, cells were stained for CD56 and CD36 and sorted by flow cytometry. Both CD56^low^CD36^+^ fraction and the remaining negative fraction were collected and cultured in fresh medium. Cells in the secondary cultures were analyzed for CD56 and CD36 at the indicated time. Shown are CD56 versus CD36 staining profiles of both fractions from one of three experiments. (**B**) CD34^+^ cells were purified from human CB and cultured in GMP differentiation medium. Fourteen days after culture, CD34^+^CD38^+^CD123^low^CD45RA^+^ GMP were purified by cell sorting and cultured in NK differentiation medium. Cells in the secondary cultures were analyzed for CD56 and CD36 at the indicated time and shown as dot plots. Shown are data from one of the two experiments. (**C**) Comparison of percentages of CD56^+^CD36^−^ cells in *in vtiro* GMP differentiation culture at day 24 and day 30. Each dot represents one independent culture. ****p < 0.0001.

To further demonstrate that the CD56^high^CD36^−^ NK cells mature through a CD56^low^CD36^+^ intermediate stage, CD56^low^CD36^+^ cells were purified by cell sorting 14 days after culture of CB CD34^+^ cells. As soon as 2 days following culture of sorted CD56^low^CD36^+^ cells in fresh medium, CD56^high^CD36^−^ cells were detected (Fig. 6). The proportion of CD56^high^CD36^−^ cells increased gradually. When the negative fraction from the cell sorting was cultured in fresh medium, newly differentiated CD56^low^CD36^+^ cells became apparent at day 4 and the proportion increased over time. As the purity of CD56^low^CD36^+^ cells was >98%, these results show that CD56^high^CD36^−^ mature NK cells are directly derived from the CD56^low^CD36^+^ precursors.

### CD56^low^CD36^+^ NK precursors are derived from GMP

We further determined whether CD56^low^CD36^+^ NK precursors are differentiated from myeloid cell progenitors. CB CD34^+^ cells were purified and cultured in differentiation medium supplemented with IL-3, IL-7, IL-15, SCF and Flt-3L. Fourteen days after culture, ~3% of the cells exhibited CD34^+^CD38^+^CD123^low^CD45RA^+^ phenotype of GMP. These cells were purified, put in the secondary culture in NK differentiation medium and monitored for CD56 and CD36 expression over time. After purification but before culture (day 0), almost all cells were negative for CD56 and CD36 (Fig. 6B). As soon as 2 days in culture, approximately 50% of cells became positive for CD36. By day 7, most cells were positive for CD36 although they were still negative for CD56. At day 14, most cells became CD56^low^. By day 24, CD56^high^CD36^−^ cells were detected and the percentage increased significantly by day 30 (Fig. 6C, 4.52% ± 0.22 vs 46.27% ± 2.97, n = 3). These results show that CD56^low^CD36^+^ NK precursors are derived from GMP.

## Discussion

**Figure 7:**
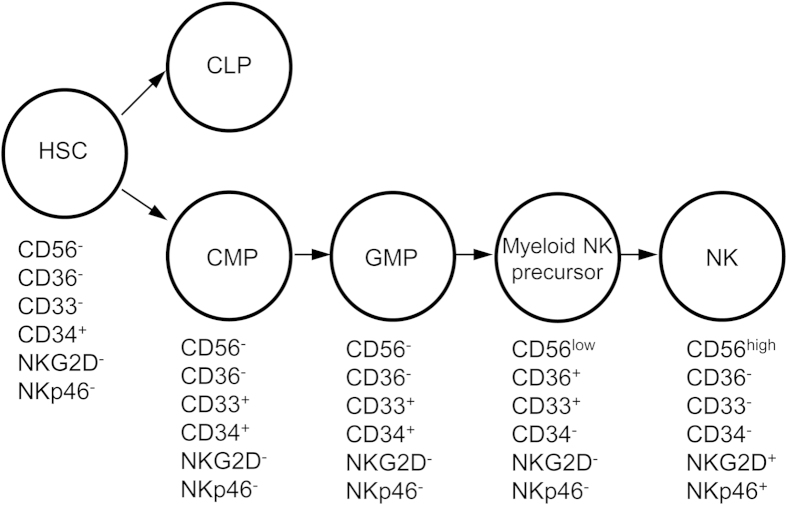
Pathway of NK cell differentiation from myeloid progenitors. Phenotypes of HSCs, CMPs, GMPs, myeloid NK precursors and NK cells are shown.

In this report, we identified an immature CD56^+^ NK cell population in the BM of humanized mice that expresses myeloid markers including CD36 and CD33 but not NK cell functional receptors such as NKG2D and NKp46. These myeloid NK precursors were also found in human CB, fetal and adult BM, suggesting their presence under normal physiological conditions in humans. By *in vitro* differentiation, we further showed that the CD36^+^CD33^+^ myeloid NK precursors are derived from CD34^+^CD38^+^CD123^low^CD45RA^+^ GMPs and give rise to mature CD56^high^ NK cells by losing expression of myeloid markers and acquiring NK cell receptors (Fig. 7). These findings help to better define differentiation pathways by which NK cells are derived from GMPs in the BM in humans.

CD56^+^ cells that express myeloid markers have been reported previously. Differentiation of human CB CD34^+^ cells into NK cells *in vitro* in the presence of IL-15, stroma cells and corticosteroids goes through an intermediate stage of CD13^+^CD33^+^ cells^9^. In the CB, a population of CD56^+^CD33^+^ NK cells exhibits a lower level of cytotoxic activity than NK cells from other organs, suggesting their immature status^10^. In addition, maturation of human NK cells in the secondary lymphoid tissues goes through a CD33^+^ stage, which is lost following maturation to CD56^high^ NK cells^11^. Our results are consistent with these previous reports and further define the pathway of NK cell differentiation from myeloid precursors. Besides expression of CD33 and CD13, our microarray analysis showed that the myeloid NK precursors express other myeloid markers, such as CD36, CSF1R, CSF2RA, FCN1 and IL13RA1, and hematopoietic progenitor markers such as FLT3 (Fig. 1B). We further show that CD36^+^CD33^+^ NK precursors are derived from CD34^+^CD38^+^CD123^low^CD45RA^+^ GMPs and differentiate into CD56^high^ NK cells by losing myeloid marker expression and acquiring NK cell functional receptors. Studies have shown that CD56^low^CD16^+^ NK cells produce more cytokines and have stronger cytotoxicity than CD56^high^CD16^−^ NK cells. The CD56^low^ myeloid NK progenitors that we described here are different from those previously described CD56^low^CD16^+^ NK cells: CD56^low^ myeloid NK progenitors do not express CD16 nor many of the functional receptors such as NKG2D and NKp46 (Fig. 1B), and have a low cytotoxic activity (Fig. 2E), whereas the CD56^low^CD16^+^ NK cells are mature and express NK cell functional receptors but not myeloid markers. Together, our findings suggest that human NK cell differentiation from myeloid progenitors in the BM goes through a CD56^low^CD33^+^CD36^+^ myeloid NK precursor stage.

Our study underscores the potential utility of the humanized mice in studying human hematopoiesis. The greatly improved human NK cell reconstitution in humanized mice following expression of human IL-15 and Flt-3L suggests that humanized mice possess the appropriate microenvironment where human NK progenitor cells can respond to the appropriate cytokines as in humans. Furthermore, identification of CD56^low^CD33^+^CD36^+^ myeloid NK precursors in the BM of humanized mice as well as in human CB, fetal and adult BM show that the development of NK cells in cytokine-treated mice goes through similar pathways as in human.

However, there are notable differences between humanized mice and humans. The proportion of CD56^low^CD33^+^CD36^+^ myeloid NK precursors is significantly higher in the BM of humanized mice than in human CB, fetal and adult BM. This could be due to the transient expression of high levels of human IL-15 and Flt-3L in humanized mice. Consistent with this explanation, humanized mice without cytokine treatment have fewer CD56^low^CD33^+^CD36^+^ myeloid NK precursors (data not shown). In addition, in the BM of humanized mice the level of CD56 on CD33^+^CD36^+^ NK precursors does not change as these cells differentiate into mature NK cells, whereas in human CB and BM CD56 is low on CD33^+^CD36^+^ NK precursors and increases as the cells differentiate into mature NK cells. Despite of these differences, the major step in the pathway of human myeloid progenitor to NK cell development appears to be the same between humanized mice and humans. Development of a small animal model where human hematopoiesis can be studied under the physiological condition should facilitate a greater understanding of hematopoiesis in human.

In addition to the study of hematopoiesis, how organ-specific NK cells play their roles in different organs is not well-known^12^, e.g. inflammation suppression by CNS-resident NK cells^16^. Murine liver NK cells were also shown to constitute a different NK cell lineage from spleen NK cells^17^. Likewise, murine lung NK cells were phenotypically different from other tissue-resident murine NK cells^18^. However, given the differences between murine and human NK cells^13^, it is important to recapitulate these findings in human NK cells. In this regard, our humanized mouse model can be an invaluable tool to study the homeostasis and functions of organ-specific NK cells.

## Materials and Methods

### Isolation of human CD34^+^ cells

Human fetal livers and femurs were obtained from aborted fetuses at 15–23 weeks of gestation in accordance with the institutional ethical guidelines of KK Women and Children Hospital, Singapore. All patients gave written informed consent for the donation of their fetal tissues for research. Fetal liver was processed as described previously^19^. Umbilical cord blood was obtained from the Singapore Cord Blood Bank. CD34^+^ cells from both fetal liver and cord blood were purified with the use of a CD34 positive selection kit (Stem Cell Technologies, Vancouver, BC); the purity of CD34^+^ cells was 90 to 99%. Viable cells were counted by Trypan Blue exclusion of dead cells. All cells were isolated under sterile conditions.

### Generation of humanized mice

NOD/scid Il2rg^−/−^ mice were obtained from the Jackson Laboratories and maintained under specific pathogen-free conditions in the animal facilities at National University of Singapore (NUS) and the Biological Resource Centre (BRC) in A*STAR, Singapore. Pups within 48 hrs of birth were sublethally irradiated (100 cGy) and engrafted with CD34^+^ fetal liver cells by intra-liver injection (2 × 10^5^ cells/recipient). Human IL-15 or Flt-3L were cloned into pcDNA3.1(^+^) vector individually as previously described^14^. Plasmid DNA was purified by Maxi-prep Kit (Promega) with endotoxin removal. For hydrodynamic injection, plasmids (50 μg each) in 1.8 ml PBS were injected into 10 weeks or older humanized mice within 7 sec using a 27-gauge needle. All experiments involving human tissues and mice were approved by the institutional committees at NUS, A*STAR and Massachusetts Institute of Technology.

### Antibodies and flow cytometry

The following antibodies from BioLegend were used for flow cytometry: anti-human CD2 (TS1/8), CD3 (HIT3a), CD10 (HI10a), CD33 (WM53), CD34 (581), CD36 (5–271), CD38 (HB-7), CD45 (H130), CD45RA (HI100), CD56 (HCD56), CD94 (DX22), CD122 (TU27), CD132 (TUGh4), CD135 (BV10A4H2), NKG2D (1D11) and NKp46 (9E2). Anti-human NKG2A antibody was from R&D systems (Minneapolis, MN). Anti-mouse CD45 (30-F11) was acquired from BD Biosciences. For flow cytometry, samples were stained with antibodies in 50 μl flow buffer (0.2% bovine serum albumin [BSA, sigma], 0.05% sodium azide [sigma] in PBS) on ice for 30 mins. Stained samples were then analyzed on a BD LSR II flow cytometer, and data analyzed with FACS Diva (BD Biosciences) and FlowJo (TreeStar version e.g. 7.6.5). Isotype-matched control antibodies were used for all fluorochrome-isotype combinations. For fluorescent-activated cell sorting (FACS), cells were stained with appropriate antibodies in RoboSep buffer (Stemcell Technologies), and sorted on a BD FACSAria II (BD Bioscience).

### MNC isolation from humanized mouse organs and human CB, fetal and adult BM

Peripheral blood, spleen, lungs, liver and BM of humanized mice 9 days post hydrodynamic injection were harvested. Lung tissue was first digested with collagenase (2 mg/ml, Gibco) for 20 mins. BM cells were flushed out of femurs using a 27G needle. Thereafter, cells from all organs were filtered through a 100 μm cell strainer (Fischer Scientific), resuspended in 40% Percoll-PBS solution, overlaid on 70% Percoll-PBS, and spun at 900 g for 20 mins. MNCs were collected at the interface between 40% and 70% Percoll. Contaminating red blood cells were lysed with ACK lysis buffer (Life Technologies). Isolation of CD56^+^ cells from MNCs was performed via cell sorting.

Human adult BM MNCs were purchased from AllCells (Alameda, CA). To isolate MNCs from fetal BM, cells were flushed out of femurs using a 27G needle. CB MNCs were separated by Ficoll-Hypaque density gradient. CD56^+^ cells were enriched by human CD56^+^ positive selection kit (StemCell Technologies) from CB, adult and fetal MNCs. CD56^+^CD36^+^ cells were purified by cell sorting.

### RNA extraction and microarray

Total RNA was isolated from purified CD56^+^ NK cells (>95% purity) from different organs with use of the RNeasy micro kit (Qiagen). Quality and quantity of RNA was determined using a Bioanalyzer (Agilent Technologies) and a nanodrop ND-1000 spectrophotometer (ThermoFisher Scientific), respectively. Transcription analysis was done with Agilent SurePrint G3 Human GE 8 × 60 K Microarray (G4858A-028004; Agilent Technologies), as per the Agilent protocol for 1-color microarray-based gene expression analysis. Data were obtained using the Agilent Feature Extraction software (version 9.5; 1-color defaults for all parameters). Data were processed using the RosettaResolver® system (version 7.2) (Rosetta Biosoftware, Kirkland, WA). Expression data from probes with annotated gene symbols were subjected to hierarchical clustering, and a sample dendrogram was produced using the R (R version 2.11.1) package pvclust (pvclust_1.2–1): this is an add-on package for the statistical software R, and is used to assess the uncertainty in hierarchical cluster analysis. Genespring GX version 11 (Agilent technologies) was used for principal component analysis.

### NK cell stimulation and cytotoxicity assay

Nine days after hydrodynamic injection of cytokine plasmids, CD56^+^ NK cells were purified from spleen and BM by cell sorting. Cells were washed and resuspended in IMDM containing 2% FCS, and cytotoxicity against the NK-sensitive target K562 (ATCC, USA) was determined in a 4-hr lactate dehydrogenase (LDH) release assay (CytoTox 96; Promega, Madison, WI).

For *in vitro* stimulation, purified NK cells were cultured in RPMI 1640, 10% FCS, 2 mM L-glutamine, 1 mM sodium pyruvate, penicillin and streptomycin at 37 °C and 5% CO_2_. 50 μg/ml poly(I:C) (Sigma) was added into the culture to stimulate NK cells *in vitro*. 24 hrs post stimulation, human IFN-γ levels in the culture supernatants were measured by ELISA (Biolegend).

### *In vitro* differentiation

For NK cell differentiation, cells were cultured in RPMI-1640 (Sigma) supplemented with 10% fetal bovine serum (FBS, Gibco), Penicillin/Streptomycin (Gibco), human IL-15 (10 ng/ml, Peprotech), Flt-3L (10 ng/ml, Peprotech) and SCF (10 ng/ml, Peprotech), at 37 °C and 5% CO_2_. For GMP differentiation, CB CD34^+^ cells were cultured in RPMI 1640 supplemented with 10% FBS, IL-3 (5 ng/mL, Peprotech), IL-7 (20 ng/mL, Peprotech), IL-15 (10 ng/mL), SCF (20 ng/mL), and Flt-3L (10 ng/mL)^9^, at 37 °C and 5% CO_2_.

### Statistical analysis

Data are presented as mean and standard error of the mean (SEM). Differences between groups were analyzed via Student’s *t-*test, with *P* value of < 0.05 considered statistically significant. All calculations were performed using the Origin 8.0 software package.

## Additional Information

**How to cite this article**: Chen, Q. *et al.* Delineation of Natural Killer Cell Differentiation from Myeloid Progenitors in Human. *Sci. Rep.* **5**, 15118; doi: 10.1038/srep15118 (2015).
